# A description of the origins, design and performance of the TRAITS–SGP Atlantic salmon *Salmo salar* L. cDNA microarray

**DOI:** 10.1111/j.1095-8649.2008.01876.x

**Published:** 2008-06

**Authors:** J B Taggart, J E Bron, S A M Martin, P J Seear, B Høyheim, R Talbot, S N Carmichael, L A N Villeneuve, G E Sweeney, D F Houlihan, C J Secombes, D R Tocher, A J Teale

**Affiliations:** * Institute of Aquaculture, University of StirlingStirling, FK9 4LA, U.K.; ‡ Scottish Fish Immunology Research Centre, School of Biological Sciences, University of AberdeenTillydrone Avenue, Aberdeen AB24 2TZ, U.K.; § School of Biosciences, Cardiff UniversityMuseum Avenue, Cardiff CF10 3US, U.K.; ¶ Norwegian School of Veterinary ScienceBasAM-Genetics, P. O. Box 8146 Dep, NO-0033 Oslo, Norway; # ARK-Genomics, Roslin InstituteRoslin, Midlothian EH 25 9PS, U. K.

**Keywords:** Atlantic salmon, DNA microarray, gene expression, immune response, lipid metabolism, smoltification

## Abstract

The origins, design, fabrication and performance of an Atlantic salmon microarray are described. The microarray comprises 16 950 Atlantic salmon-derived cDNA features, printed in duplicate and mostly sourced from pre-existing expressed sequence tag (EST) collections [SALGENE and salmon genome project (SGP)] but also supplemented with cDNAs from suppression subtractive hybridization libraries and candidate genes involved in immune response, protein catabolism, lipid metabolism and the parr–smolt transformation. A preliminary analysis of a dietary lipid experiment identified a number of genes known to be involved in lipid metabolism. Significant fold change differences (as low as 1·2×) were apparent from the microarray analysis and were confirmed by quantitative real-time polymerase chain reaction analysis. The study also highlighted the potential for obtaining artefactual expression patterns as a result of cross-hybridization of similar transcripts. Examination of the robustness and sensitivity of the experimental design employed demonstrated the greater importance of biological replication over technical (dye flip) replication for identification of a limited number of key genes in the studied system. The TRAITS (TRanscriptome Analysis of Important Traits of Salmon)–salmon genome project microarray has been proven, in a number of studies, to be a powerful tool for the study of key traits of Atlantic salmon biology. It is now available for use by researchers in the wider scientific community.

## Introduction

The Atlantic salmon (*Salmo salar* L.) is an important farmed fish species throughout its native range (western Europe and east coast North America). Over the past 10 years, a substantial Atlantic salmon mariculture industry has also been established in Chile. In indigenous areas, the wild species also underpins valuable sectors of the rural economy founded on sport and ecotourism. Its biology is unusual in that, as an anadromous species, it adapts to very different environments in terms of temperature and salinity at different stages in its life cycle. Atlantic salmon is a high-value food source providing quality protein and oils, and together with other oily fish, it is the most important source of essential omega-3 fatty acids in the human diet. Data compiled from FAO UN database (FishStat Plus) indicate that farmed Atlantic salmon production in the world exceeds 1·2 million tonnes per annum.

**Fig. 1:**
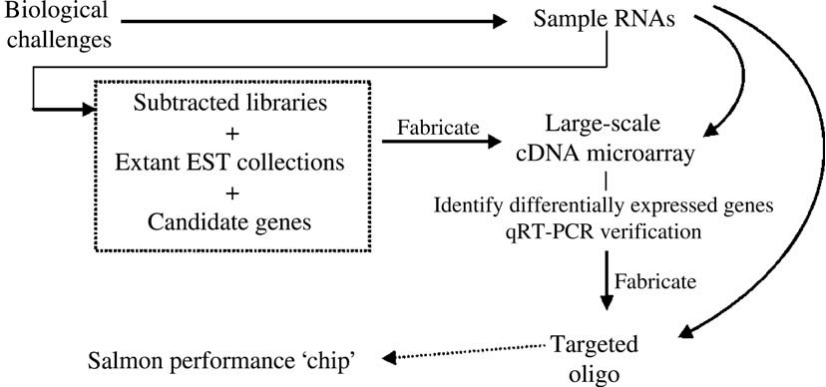
Overview of TRanscriptome Analysis of Important Traits of Salmon strategy to generate both a general cDNA and a focused oligonucleotide microarray. qRT-PCR, quantitative real-time polymerase chain reaction analysis. EST, expressed sequence tag; qRT-PCR quantitative real-time polymerase chain reaction.

Currently, the four most important constraints on commercial production of this species are (a) supply of dioxin-free highly unsaturated oils for the salmon diet, (b) protein growth efficiency, (c) infectious disease and (d) a long and complex life cycle. In 2002, research groups from three U.K. universities (Aberdeen, Cardiff and Stirling), specializing in different aspects of Atlantic salmon biology, formed a partnership to develop and exploit transcriptomic resources to explore the molecular basis of the biology underlying these constraints. The goal of TRAITS (TRanscriptome Analysis of Important Traits of Salmon, http://www.abdn.sfirc/salmon) was to bolster the sustainability of Atlantic salmon farming through identification of genes and metabolic pathways influencing traits that are important in terms of (a) efficiency and sustainability of farm production, (b) welfare of farmed stocks and (c) quality and nutritional value of salmon products for the consumer. This goal was to be achieved through selection of a set of key indicator genes associated with the traits of interest, in order to form the basis of a prototype DNA chip for monitoring salmon health and performance. The underlying strategy (Fig. 1) was to design a primary cDNA microarray based on extant expressed sequence tag (EST) collections together with novel ESTs drawn from subtracted libraries associated with targeted laboratory and, or field ‘challenges’. RNA samples derived from these and other challenges would be interrogated by the cDNA array to identify candidate responder genes and gene pathways. A second more focused oligonucleotide array, comprising mainly responder genes, would then be fabricated and initially validated by interrogation of the same samples that were hybridized to the cDNA array.

The development of the TRAITS cDNA microarray was initially reliant upon a *c.* 11 k EST collection from the European Union (EU)-funded SALGENE project (‘Construction of a genetic body map for Atlantic salmon’; FAIR CT98 4314), in which Stirling had been a partner. However, prior to project start up, a formal collaboration with the Norwegian Salmon Genome Project (SGP, http://www.salmongenome.no) was developed that allowed access to a more extensive physical EST resource (Hagen-Larsen *et al.*, 2005; Adzhubei *et al.*, 2007). The TRAITS–SGP cDNA microarray described in this paper is the product of this collaboration.

Several cDNA microarray platforms have been established for salmonid fish with varying numbers of features: 1380 features (Koskinen *et al.*, 2004), 3700 features (Rise *et al.*, 2004), 4104 features (Ewart *et al.*, 2005), 79 features (Jordal *et al.*, 2005) and 16 008 features (von Schalburg *et al.*, 2005). A number of different biological processes have been examined by microarray in salmonid fish. These include immune responses to bacterial infections (Rise *et al.*, 2004; Ewart *et al.*, 2005; von Schalburg *et al.*, 2005; MacKenzie *et al.*, 2006; Martin *et al.*, 2006; Wynne *et al.*, 2008), viral infections (Purcell *et al.*, 2006) and fungal infections (Roberge *et al.*, 2007). Aspects of physiology have also been examined including nutritional states (Jordal *et al.*, 2005), mobilization of energy reserves (Salem *et al.*, 2006) and stress responses (Cairns *et al.*, 2008). In the above studies, varying complexities of experimental design were employed, using different numbers of replicates and often only genes showing two-fold or higher differences in expression were selected for further scrutiny and, or characterization.

It is widely accepted that the supply of fish meal and oils for the diets of farmed fish is not sustainable (Sargent & Tacon, 1999). One consequence of this has been an increased emphasis on the development of diets based on vegetable oil (VO), which are suitable for aquaculture. A key aspect of salmonid lipid metabolism that is being extensively investigated in this regard is the conversion of C_18_ polyunsaturated fatty acids (PUFAs), found in VO, to the C_20_ and C_22_ highly unsaturated fatty acids (HUFAs), eicosapentaenoate and docosahexaenoate, which are the specific omega-3 fatty acids responsible for the health-promoting properties of fish and fish oil (FO). These components are critical to the maintenance of nutritional quality of farmed fish. Two key enzymes involved in this pathway, Δ5 and Δ6 fatty acyl desaturase (FAD), have been characterized in depth (Hastings *et al.*, 2004; Zheng *et al.*, 2005*a*). Both these genes have been shown, by quantitative real-time polymerase chain reaction analysis (qRT-PCR), to exhibit diet-dependent differential gene expression (Zheng *et al.*, 2004, 2005*a*, *b*), although detected fold change differences are minimal (1·3–2·0).

In this paper, design and fabrication details relating to the TRAITS–SGP cDNA microarray are reported. Results of a preliminary transcriptomic analysis from a dietary lipid feeding trial are used to explore both the robustness and the sensitivity of analysis that may be achieved by the use of this microarray.

## Materials and methods

### cDNA resources

#### Archived ESTs

Two main EST collections (SALGENE *c.* 11 k clones and SGP *c.* 30 k clones) were available to the TRAITS consortium in 2004. In all cases, fish were sourced from farm stocks of European origin, and library construction began with mRNAs from tissues being used as template for oligo(dT)-primed reverse transcription. The SALGENE resource comprised ESTs from seven tissue-specific non-normalized libraries and two tissue-specific normalized libraries, with all cDNAs being directionally cloned into vectors. Details of tissues used, life-history stage (juvenile, *i.e.* freshwater phase, or adult, *i.e.* marine phase) and cloning systems employed are given in Table I. Non-normalized library construction has been detailed elsewhere (Martin *et al.*, 2002; Hagen-Larsen *et al.*, 2005; Adzhubei *et al.*, 2007). Insert size varied among libraries but ranged from 300 base pairs (bp) to 4 kbp. Single-pass sequence data (5′-end) were available for all clones. Normalized libraries were made in M. B. Soares’ laboratory, University of Columbia, U.S.A., following their standard methodology (Bonaldo *et al.*, 1996). Single-pass sequence data available for these clones were a mixture of both 5′- and 3′-end reads. SGP clones were derived from 14 tissue-specific non-normalized libraries: brain, eye, gill, head kidney, heart, intestine, kidney, liver, white muscle, ovary, skin, spleen, swimbladder and testis. All tissues were sampled from parr (freshwater phase). The cDNAs were directionally cloned into pBlueScript II SK(+) XR phagemid vector and transformed into XL10-Gold host cells (Hagen-Larsen *et al.*, 2005; Adzhubei *et al.*, 2007). EST data comprised single-pass 5′-end sequences.

**Table I:** Details of the expressed sequence tag (EST) libraries used to construct the TRAITS–SGP cDNA microarray

Source	Tissue	Environment	Host cells	Vectors
SALGENE	Liver, testis and ovary	Sea water	XLOLR	pBK-CMV
SALGENE	Spleen and kidney	Sea water	SOLR	pBlueScript II SK(−)
SALGENE	Gill and intestine	Fresh water	XL10-Gold	pBlueScript II SK(+) XR
SALGENE	White muscle and brain	Sea water	SURE	pT7T3-Pac
TRAITS	Liver, kidney, gill and white muscle	Fresh water	ElectroTen; Blue	pGEM T-easy
TRAITS	Liver, kidney, brain, pituitary and gill	Sea water	ElectroTen; Blue	pGEM T-easy
SGP	Brain, eye, gill, head kidney, heart, intestine, kidney, liver, white muscle, ovary, skin, spleen, swimbladder and testis	Fresh water	XL10-Gold	pBlueScript II SK(+) XR

SGP, salmon genome project; TRAITS, TRanscriptome Analysis of Important Traits of Salmon.

#### Trait-specific enriched libraries

All enrichments used a standard approach – suppression subtractive hybridization (SSH, PCR-Select cDNA Subtraction Kit; Clontech, Mountain View, CA, U.S.A.). Non-directional cloning was subsequently used to insert subtracted cDNA fragments into the pGEM T-Easy vector (Promega, Madison, WI, U.S.A.). Resultant cDNA fragments generally ranged in size between 150 and 700 bp.

#### Pathogen-induced libraries

A bacterial challenge was undertaken using *Aeromonas salmonicida*, the bacterial pathogen responsible for furunculosis of salmon. Three tissue-specific enriched libraries (head kidney, gill and liver) were constructed (Martin *et al.*, 2006). Fish were anaesthetized with benzocaine (20 mg l^−1^; Sigma-Aldrich, St Louis, MO, U.S.A.) and injected intraperitoneally with 100 μl (10^9^ CFU ml^−1^) of a genetically attenuated strain (aroA^−^) of *A*. *salmonicida*(Brivax II; Sigma-Aldrich) (Marsden *et al.*, 1996) in phosphate-buffered saline (PBS) or 100 μl of PBS as control. Brivax II is a non-virulent strain but acts in a similar manner to the intact virulent pathogen; however, after several rounds of replication, the fish clears the bacteria. Intraperitoneal injection of Brivax II induces a protective immune response, with fish resistant to a later challenge with virulent strains. The two groups of fish were kept separately and RNA was pooled from 10 challenged fish and 10 control fish at 24 and 48 h after injection. In each case, the challenged RNA was ‘tester’ and the control ‘driver’. Approximately 500 clones from each library were sequenced, a mean redundancy of *c.* 33% being observed. Following basic local alignment search tool (BLAST)X sequence homology matching, 20, 23 and 50% of genes had sequences homologous to immune-associated genes for head kidney, gill and liver, respectively (Martin *et al.*, 2006).

#### Starvation-induced libraries

Two tissue-specific enriched libraries (white muscle and liver) were constructed following a starvation trial. RNA was pooled from 10 fish starved for 14 days and from 10 fish fed *ad libitum*. RNA from the starved pool was used as ‘tester’ and RNA from fed fish as ‘driver’. For genes enriched following short-term starvation, a highly heterogeneous group of genes was found, as many different biological processes were altered by this treatment including those related to protein turnover. For the library generated from liver, 92% of the sequences were found to have homologies following BLASTX searches. Key groups of genes represented in the library-encoded metabolic enzymes, serum proteins and immune response genes, with other minor groupings being iron-binding proteins, globins and factors involved in transcription and translation. For the genes enriched in muscle following starvation, 77% had BLASTX homologies, with key groups of sequences encoding metabolic enzymes, structural proteins and transcription and translation factors (10%) and minor groups including heat shock proteins.

#### Diet response libraries

Atlantic salmon were fed from first feeding on diets containing either FO (capelin oil) or a 25% FO:75% blended VO diet (see *Feed Trial*). Four subtracted liver cDNA libraries [two time-points – 52 weeks (pre-smolt–fresh water) and 55 weeks (post-smolt–sea water) and two directions, FO driver and VO driver] were made. Pooled RNA from 12 (pre-smolt) and four (post-smolt) female fish on each diet was used. A total of 768 clones were sequenced. All four libraries were found to be highly redundant; 10 fragments comprised *c.* 40% of all sequences. BLASTX analyses gave significant hits (e value < e^−20^) for 79 (54%) of the 145 different sequences, although only one of these (catfish fatty acid-binding protein) appeared to be directly related to lipid metabolism.

#### Smoltification response libraries

Four tissue-specific SSH libraries enriched in genes upregulated in brain, pituitary, kidney and gill of smolts (seawater phase) in comparison to parr (freshwater phase) were made. Tissues were dissected from parr and smolt in November–December 2002 and April–May 2003. A total of 380 clones from each of the four libraries were sequenced. Between 32 and 50% of the sequenced clones were identified by BLASTX sequence homology searches. All libraries had considerable redundancy (28% brain, 56% gill, 56% kidney and 86% pituitary). Significantly, the single most abundant sequence in the gill subtractive library corresponded to Na^+^ and K^+^-ATPase whose levels of activity are a key indicator of smoltification status.

#### Candidate and other genes

A third, minor source of cDNAs for the array was a small collection of full-length genes or gene fragments in plasmid constructs that were already possessed by the partners. These included both candidate genes and also other genes with no known relevance to the specific traits of interest (Table II). Being better characterized than the EST clones, these constructs were potentially useful as reference genes on the microarray. Of note, with regard to the research reported in this paper, is the inclusion of three cDNA fragments [the open reading frame (ORF) and two 3′-untranslated region (UTR) fragments] from both Atlantic salmon FAD genes (Δ5 FAD and Δ6 FAD).

**Table II:** Pre-identified candidates and other reference genes

Gene name	cDNA lengths (bp)	Notes
Apolipoprotein B	1402	Partial with 3′-UTR
Carnitine palmitoyltransferase 1	823	Partial
Carotene dioxygenase	872	Partial
Oestrogen receptor-alpha	2900	Partial with 3′-UTR
Growth hormone receptor	340	Partial, PCR fragment
Glyceraldehyde phosphate dehydrogenase	1086	Full length
Heat shock protein P70	830	Partial with 3′-UTR
Homogentisate dioxygenase	952	Partial with 3′-UTR
Insulin-like growth factor-1	230	Partial
Interferon-gamma	1132	Full length
Interleukin-1 beta	790	ORF
NGF1-B	224	Partial (RACE fragment)
Pituitary-specific transcription factor 1	250	Partial
Peroxisome proliferator-activated receptor-alpha	1644	Full length
Peroxisome proliferator-activated receptor-beta 1	1462	Full length
Peroxisome proliferator-activated receptor-beta 2	779	Partial
Peroxisome proliferator-activated receptor-gamma	1665	Full length
Polyunsaturated fatty acid elongase	950	ORF
Retinoic acid receptor-alpha	840	ORF (RACE fragment)
Retinoic acid receptor-gamma	440	Partial inc 5′-UTR
Retinaldehyde dehydrogenase type 2	922	Partial
TNF-α induced adipose related protein	483	Partial
Thyroid hormone receptor-alpha	*c.* 2000	Partial inc 5′-UTR
Thyroid hormone receptor-beta	*c.* 900	Partial inc 5′-UTR
Vitamin D3 receptor	360	Partial inc 5′-UTR
Δ5 FAD	408	3′-UTR fragment
Δ5 FAD	881	3′-UTR fragment
Δ5 FAD	1365	ORF
Δ6 FAD	384	3′-UTR fragment
Δ6 FAD	401	3′-UTR fragment
Δ6 FAD	1365	ORF

FAD, fatty acid desaturase; inc, including; NGF, neuronal growth factor; ORF, open reading frame; PCR, polymerase chain reaction; RACE, rapid amplification of cDNA ends; TNF, tumour necrosis factor; UTR, untranslated region.

### Sequence clustering and probe selection

**Fig. 2:**
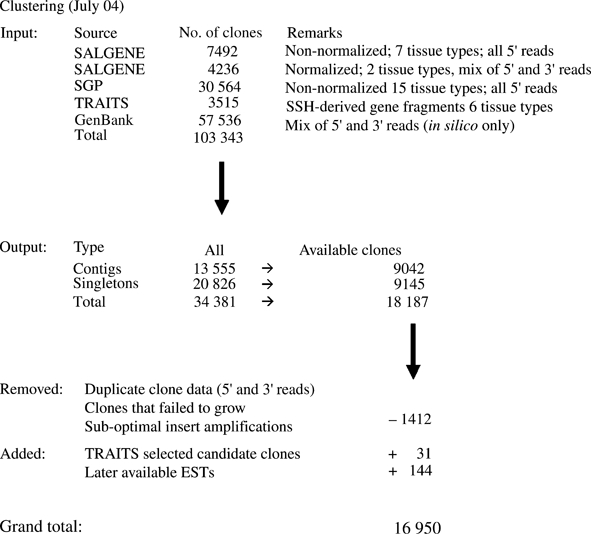
Summary of clustering procedure and probe selection for the cDNA microarray. SGP, salmon genome project; TRAITS, TRanscriptome Analysis of Important Traits of Salmon.

All sequence data derived from the above resources, together with 57 k Atlantic salmon sequences available *in silico* from GenBank (National Center for Biotechnology Information–NCBI) in July 2004, were clustered using the The Institute of Genomic Research (TIGR) gene indices (TGI) clustering tools (Pertea *et al.*, 2003). The process of clone selection for the cDNA microarray is summarized in Fig. 2. Approximately equal numbers (*c.* 9 k) of contigs and singletons were identified as having accessible clones. Contig sequence lengths varied from 135 to 3804 bp (mean 956 bp) and comprised from 2 to 651 clone sequences (mean 9·1 sequences per contig). Because the overall number (*c.* 18 k potential features) was comfortably within the printing capacity of the microarray spotter, no further clone selection or refinement was undertaken. A single representative clone from each contig was selected for inclusion on the microarray. The only selection criteria applied were (a) where possible, a SALGENE clone was selected in preference to an SGP clone because the entire SALGENE resource was archived at the printing site (ARK Genomics, Roslin Institute, U.K.) and (b) clones were selected from non-normalized or normalized libraries in preference to SSH-derived cDNAs in order to take advantage of longer transcripts. Within this defined sub-set, a clone was selected at random for inclusion on the array. There was no intentional selection of a 5′- or 3′-biased clone from within each contig.

### Microarray fabrication

Bacterial cultures were grown from archived samples and cDNA inserts amplified directly from these using vector-specific primers. Initially, various primer sets were used, dependent on the specific vector employed. However, a generic set (BSKS-F: CGATTAAGTTGGGTAACGC and BSKS-R: CAATTTCACACAGGAAACAG) was found to work with all but one vector. For pBK-CMV constructs, T3 (AATTAACCCTCACTAAAGGGA) and T7 (TAATACGACTCACTATAGGG) primers were employed. PCR reactions (50 μl) were purified by passing them through a 384-well multiscreen filter plate (Millipore, Watford, U.K.). Amplicons were electrophoresed through a 1% agarose gel and stained with ethidium bromide. Only PCR products producing a clear singlet band were considered for spotting. Amplicons were quantified using PicoGreen assay (Invitrogen, Madison, WI, U.S.A.). Features were printed onto aminosilane-coated glass slides (Corning GAPS II; Corning Inc., Corning, NY, U.S.A.) using a MicroGrid II printer (Genomic Solutions, Holiston, MA, U.S.A.). DNA was resuspended in printing buffer (150 mM sodium phosphate buffer, 0·1% sodium dodecyl sulphate (SDS), pH 8·0) to a concentration of *c.* 150 ng ml^−1^and spotted with a 48 pin tool (Biorobotics 10 k pins; Biorobotics Ltd, Cambridge, U.K.). Mean spot diameter was 110 μm. The slide format comprised 48 sub-arrays, each consisting of 27 columns and 28 rows. Each cDNA was printed in duplicate, with duplicate features being printed non-adjacently within the same sub-array. In addition to these 16 950 Atlantic salmon cDNAs, a number of control features were printed across the microarray to maximize its flexibility in terms of possible experimental applications. These control features comprised sonicated Atlantic salmon genomic DNA (96 features), sheared salmon sperm DNA (*Oncorhynchus* sp. derived – Sigma; 96 features), four different SpotReport® (Stratagene, La Jolla, CA, U.S.A.) controls – namely PCR products 1–3 (Cab, RCA and rbcL genes from *Arabidopsis thaliana*) and human β-actin PCR fragment (20 features each) and spotting buffer (192 features). In addition, each sub-array had two Cy3 spots (landing lights) located at the upper left corner to aid orientation of the slide during grid placement and spot finding procedures. The remaining 1826 locations on the 36 288 spot grid were left blank. Following printing, DNA spots were fixed by baking at 80° C for 2 h. Prior to hybridization, microarray slides were treated using succinic anhydride and 1-methyl-2-pyrrolidinone (Sigma) to block unbound amino groups (slide manufacturer's recommended protocol) and denatured by incubation at 95° C in MilliQ water for 2 min. Slides were then rinsed twice in isopropanol, centrifuged to dry and stored in a desiccated environment until required. Details of the TRAITS cDNA microarray have been submitted to the ArrayExpress platform (http://www.ebi.ac.uk/arrayexpress) under accession number A-MEXP-664. The GAL file is also available for inspection from the TRAITS–SGP microarray website (http://www.traitsdb.stir.ac.uk/). All clones used on the microarray are archived in 384-well plates as glycerol stocks in two locations (ARK Genomics Facility, Roslin Institute, U.K., and SGP Genetics Laboratory, University of Oslo).

### EST annotation

Gene identification was carried out by BLAST searches (Altschul *et al.*, 1990) of appropriate databases, (NCBI) non-redundant (nr) nucleotide and protein databases, and interrogation of the TIGR Atlantic salmon Gene Index, release 3.0 (http://biocomp.dfci.harvard.edu/tgi/cgi-bin/tgi/gimain.pl?gudb=salmon). Gene ontology (GO) identifiers were obtained through Blast2GO (Conesa *et al.*, 2005).

### Sensitivity of microarray protocol

#### Feed trial

The effect of replacing FO with VO at a replacement level of 75% was investigated in Atlantic salmon in a trial conducted over an entire 2 year production cycle (Torstensen *et al.*, 2005). Briefly, the two diets were fed to triplicate tanks and cages at Marine Harvest Ltd., facilities at Invergarry (fresh water) and Loch Duich, Lochalsh (sea water), U.K. Atlantic salmon fry were distributed randomly into six tanks (3 m × 3 m, depth 0·5 m) at a stocking level of 3000 per tank and weaned onto extruded feeds containing 20% added oil, which was either FO (capelin oil) or a VO blend, containing rapeseed, palm and linseed oils in a 3·7:2:1 ratio, replacing 75% of the FO. This VO blend was formulated to mimic FO in saturated and monounsaturated fatty acid content but with C_18_ PUFA replacing n-3 HUFA. Fish were fed the diets described above for 1 year until seawater transfer, at which point fish (mean mass *c.* 50 g) were transferred into 5 m × 5 m net pens at 700 fish pen^−1^. The fish were fed the same diet in sea water as in fresh water although the dietary oil levels were increased to 25% (3 mm pellet), rising to 32% (9 mm pellets) through the year-long seawater phase. The diets aimed to be practical and were formulated and manufactured by Skretting ARC (Stavanger, Norway) according to current practices in the salmon-feed industry. All diets were formulated to satisfy the nutritional requirements of salmonid fish (NRC, 1993).

#### Sample preparation

Fish fed on each diet were sampled at two time-points during freshwater rearing (at 36 and 52 weeks post-hatch, the latter just 1 week before transfer to sea) and at a further two time-points in sea water (at 55 and 86 weeks post-hatch). Twenty-four liver samples per dietary treatment and time-point were collected. Total RNA was isolated by organic solvent extraction (TriReagent; Sigma) following the manufacturer's protocol. Spectrophotometry (Nanodrop, Wilmington, DE, U.S.A.) and electrophoresis (Bioanalyser 2100; Agilent Technologies, Santa Clara, CA, U.S.A.) were used to quantify and assess the quality of the RNAs, respectively. For the transcriptomic analysis, equal amounts of RNA from four individuals (two males + two females) were pooled to produce six biological replicates per diet per time-point. Each pooled RNA sample was further cleaned by mini spin-column purification (RNeasy; Qiagen, Valencia, CA, U.S.A.) and was re-quantified and quality assessed as above.

#### Experimental design

Each biological replicates was co-hybridized in a two-dye experiment with a single pooled reference sample. This design permits valid statistical comparisons across both diets and time-points to be made. The pooled reference sample comprised equal amounts of RNA from each of the 48 biological replicate samples. A dye-swap procedure was incorporated to mitigate selective binding and scanning artefacts. Thus, the entire experiment comprised 96 separate hybridizations (two diets × four time-points × six biological replicates × two dye swaps).

#### Labelling and hybridization protocols

Because of the large number of hybridizations, not all hybridizations could be completed at the same time. Samples were therefore randomized and processed in two batches 1 week apart. RNA was reverse transcribed and labelled with either Cy3 or Cy5 fluors using the FAIRPLAY II cDNA indirect labelling kit (Stratagene) according to the manufacturer's instructions. Briefly, 20 μg total RNA was reverse transcribed after being primed with oligo dT, which incorporated aminoallyl-dUTP into the synthesized cDNA strand. The RNA template was then hydrolysed using 1 M NaOH for 15 min and neutralized with 1 M HCl. The cDNA was NaAce–ethanol precipitated overnight. cDNA pellets were washed in 80% ethanol and air-dried before being resuspended in 5 μl of 2 × coupling buffer. Once the cDNA had fully dissolved (after at least 30 min), 5 μl of either Cy3 or Cy5 dye was added and the samples were incubated in the dark for 30 min. The Cy3 and Cy5 dyes (Amersham Pharmacia, Little Chalfont, U.K.) were dissolved in 45 μl dimethyl sulphoxide (DMSO) prior to being added to the coupling buffer. To remove unincorporated dye, the labelled cDNA (total volume 10 μl) was passed through a SpinEX column (Qiagen). Dye incorporation was assessed by separating 1 μl of the sample on a mini agarose gel and visualizing fluorescent products on a microarray scanner (Perkin Elmer ScanArray 5000XL; Perkin-Elmer, Wellesley, MA, U.S.A.). No pre-hybridization step was required. For hybridization, the remainder of each labelled cDNA (7–9 μl, 16–30 pmol each dye) was added to 85 μl hybridization buffer (UltraHyb; Ambion; Austin, TX, U.S.A.), 10 μl poly(A)_80_ (10 mg ml^−1^; Sigma) and 5 μl ultrapure BSA (10 mg ml^−1^; Ambion). The hybridization mixture was heated to 95° C for 3 min, then cooled to 60° C before being applied to the microarray. Hybridizations (48 slides per day) were performed on a Gene TAC Hyb Station (Genomic Solutions) for 16 h at 45° C. Slides were then automatically washed with 2 × SSC and 0·5% SDS for 10 min at 60° C, 0·2 × SSC and 0·5% SDS for 10 min at 42° C and finally 0·2 × SSC for 10 min at 42° C. Slides were then manually rinsed in isopropanol and dried by centrifugation before being scanned.

#### Data acquisition and analysis

Hybridized slides were scanned at 10 μm resolution using a Perkin Elmer ScanArray Express HT scanner. BlueFuse software (BlueGnome, Cambridge, U.K.) was then used to identify features and extract fluorescence intensity values from the resultant TIF images. Following a manual spot removal procedure and fusion of duplicate spot data (BlueFuse proprietary algorithm), the resulting fluorescence intensity data and quality annotations for the 16 950 gene features were exported into the GeneSpring GX version 7.3.1 analysis platform (Agilent Technologies). All control features (positive, negative, landing lights, *etc.*) were excluded from subsequent analyses. Data transformation, normalization and quality filtering were as follows: (a) all intensity values <0·01 were set to 0·01, (b) a ‘per spot per chip’ intensity-dependent (Lowess) normalization was undertaken using software defaults (20% smoothing/cutoff 10) and (c) data were filtered using a BlueFuse spot confidence value >0.1 in ≥24 slides and BlueFuse spot quality of ≥0·5 in ≥24 slides. This gave a final list of 11 800 genes that were eligible for statistical analysis. Experimental annotations complied fully with minimum information about a microarray experiment (MIAME) guidelines (Brazma *et al.*, 2001). The experimental hybridizations are archived on the EBI ArrayExpress database (http://www.ebi.ac.uk/arrayexpress/) under accession number E-TABM-449.

Hybridization data were analysed by two-way ANOVA, which examined the explanatory power of the variables ‘time-point’ and ‘diet’ and the interaction between the two and incorporated a Benjamini & Hochberg (1995) multiple test correction (*P* ≤ 0·05).

#### qRT-PCR validation

Quantitative real-time-PCR was performed as described by Villeneuve *et al.* (2005). Primer details are given in Table III. Relative expression ratios were statistically compared between diet samples following normalization against three housekeeping genes, using REST software (Pfaffl, 2001; Pfaffl *et al.*, 2002). Two of the three housekeeping genes (β-actin and elongation factor-1α) are widely used as reference genes for salmonid quantitative PCR (qPCR) studies (Olsvik *et al.*, 2005). From the microarray analysis, a third reference was selected, an anonymous cDNA feature on the TRAITS–SGP microarray that was identified as a flatliner both between diets and over all time-points. Differences in gene expression between diet samples were evaluated in group means using randomization tests (REST software), which employed 5000 random allocations and considered differences to be significant at *P* < 0·05.

**Table III:** Primer details for both lipid metabolism and control genes used in quantitative real-time polymerase chain reaction assay. Annealing temperature of 60° C was used in all cases

Gene identity	Primer 1 (5′ → 3′)	Primer 2 (5′ → 3′)
Lipid metabolism genes		
Δ5 FAD (3′-UTR)	GTGAATGGGGATCCATAGCA	AAACGAACGGACAACCAGA
Δ6 FAD (3′-UTR)	CCCCAGACGTTTGTGTCAG	CCTGGATTGTTGCTTTGGAT
Lathosterol oxidase	CACTAACCTTATTCCATCGGCTACTT	TTTCCCTTCCTTTTACAGACATCAAT
Squalene monooxygenase	TGATCTCGGCTACTTTTTGTTTTTG	GCCGCCAGGATTATCTCTTTGT
Isopentenyl-diphosphate Δ-isomerase*	ACAGCCCTATGGTTATGTGTCATCTC	CAAGGTGAGGCGAATGTTTGAAC
‘Housekeeping’ reference genes		
β-actin	ACATCAAGGAGAAGCTGTGC	GACAACGGAACCTCTCGTTA
Elongation factor-1α	CTGCCCCTCCAGGACGTTTACAA	CACCGGGCATAGCCGATTCC
Unidentified liver EST*	AGCCTATGACCAACCCACTG	TGTTCACAGCTCGTTTACCG

FAD, fatty acyl desaturase; EST, expressed sequence tag; UTR, untranslated region.

* Identified as a flatliner from microarray analysis of same sample set.

## Results

### Microarray feature overview

cDNAs derived from 15 different tissue sources are represented among the 16 950 Atlantic salmon gene features printed on the TRAITS–SGP cDNA microarray (Table I). Of these, *c.* 9% are from SSH libraries. Brain cDNAs predominate (15% of total). This reflects the large number of ESTs generated for this tissue because of (a) brain libraries being included in all three of the EST resources available, (b) increased sequencing effort being focused on these libraries due to perceived diversity of gene expression within brain tissue and (c) availability of a normalized brain EST library. BLASTX homology searches of contig and singleton sequences (Table IV) revealed close to 40% of features having a weak hit (e value ≥ e^−10^) or no hit at all to the NCBI nr protein database. Among the 10 399 features with a significant BLASTX hit (e value < e^−10^), 6762 (65%) nominally different genes were identified. GO annotations were obtained for 7749 features. The most prevalent GO annotations on the microarray are summarized in Table V. Individual feature annotations can be accessed from the TRAITS–SGP microarray website (http://www.traitsdb.stir.ac.uk/).

**Table IV:** Summary of expressed sequence tag (EST) tissue representation and BLASTX analyses for the cDNA microarray

			BLASTX hits (NCBI nr protein database January 2007)		
			
Tissue	Number of ESTs	SSH derived (%)	e values < e^−40^ (%)	e values < e^−10^ (%)	e value ≥ e^−10^/no hit (%)
Brain	2524	7	44	62	38
Eye	1609	—	28	44	56
Gill	1440	24	37	60	40
Head kidney	801	—	40	58	42
Heart	1111	—	40	58	42
Intestine	1140	—	47	71	29
Kidney	1400	28	41	60	40
Liver	720	42	56	78	22
White muscle	1621	14	48	68	32
Ovary	1432	—	45	66	34
Pituitary	79	100	20	34	66
Skin	134	—	40	59	41
Spleen	1275	—	35	57	43
Swimbladder	287	—	34	51	49
Testis	1299	—	44	65	35
Unknown	47	—	39	58	37
Totals	16919	9	41	61	39

BLAST, basic local alignment search tool; EST, expressed sequence tag; NCBI, National Center for Biotechnology; nr, non-redundant; SSH, suppression subtractive hybridization.

**Table V:** The top 60 GO terms by prevalence from both biological and molecular ontologies (level 3) represented on the TRAITS–SGP cDNA microarray (significant BLASTX hit defined as e value < e^−10^)

GO ontology: biological process-level 3		
	
GO term	GO description	Features*
GO:0044237	Cellular metabolic process	3693
GO:0044238	Primary metabolic process	3489
GO:0043170	Macromolecule metabolic process	2904
GO:0050789	Regulation of biological process	1526
GO:0050794	Regulation of cellular process	1354
GO:0006810	Transport	1215
GO:0016043	Cell organization and biogenesis	1159
GO:0007154	Cell communication	1148
GO:0009058	Biosynthetic process	1030
GO:0019222	Regulation of metabolic process	903
GO:0006091	Generation of precursor metabolites and energy	571
GO:0009056	Catabolic process	468
GO:0006807	Nitrogen compound metabolic process	446
GO:0048869	Cellular developmental process	431
GO:0051641	Cellular localization	426
GO:0051649	Establishment of cellular localization	423
GO:0007275	Multicellular organismal development	404
GO:0048856	Anatomical structure development	404
GO:0045184	Establishment of protein localization	385
GO:0048468	Cell development	357
GO:0006950	Response to stress	340
GO:0016265	Death	312
GO:0007049	Cell cycle	310
GO:0022402	Cell cycle process	246
GO:0008283	Cell proliferation	219
GO:0042221	Response to chemical stimulus	216
GO:0007155	Cell adhesion	210
GO:0009653	Anatomical structure morphogenesis	194
GO:0050877	Neurological process	182
GO:0009605	Response to external stimulus	182
GO:0046903	Secretion	169
GO:0006955	Immune response	157
GO:0065009	Regulation of a molecular function	152
GO:0006952	Defence response	131
GO:0006928	Cell motility	130
GO:0051674	Localization of cell	130
GO:0009607	Response to biotic stimulus	102
GO:0009719	Response to endogenous stimulus	95
GO:0051239	Regulation of multicellular organismal process	92
GO:0019725	Cell homeostasis	79
GO:0006936	Muscle contraction	78
GO:0065008	Regulation of biological quality	72
GO:0015976	Carbon utilization	66
GO:0016049	Cell growth	61
GO:0040008	Regulation of growth	61
GO:0051301	Cell division	60
GO:0002252	Immune effector process	56
GO:0019748	Secondary metabolic process	54
GO:0007610	Behaviour	48
GO:0050878	Regulation of body fluids	48
GO:0019953	Sexual reproduction	47
GO:0000051	Urea cycle intermediate metabolic process	45
GO:0002682	Regulation of immune system process	41
GO:0051707	Response to other organism	40
GO:0002253	Activation of immune response	40
GO:0040012	Regulation of locomotion	31
GO:0050793	Regulation of developmental process	33
GO:0009628	Response to abiotic stimulus	36
GO:0048646	Anatomical structure formation	36
GO:0050817	Coagulation	38
GO ontology: molecular function-level 3		
GO:0005515	Protein binding	2223
GO:0003676	Nucleic acid binding	1293
GO:0043167	Ion binding	1150
GO:0016787	Hydrolase activity	1122
GO:0000166	Nucleotide binding	1051
GO:0016740	Transferase activity	674
GO:0016491	Oxidoreductase activity	584
GO:0004871	Signal transducer activity	462
GO:0003735	Structural constituent of ribosome	261
GO:0003700	Transcription factor activity	240
GO:0004857	Enzyme inhibitor activity	175
GO:0016874	Ligase activity	169
GO:0008289	Lipid binding	145
GO:0048037	Cofactor binding	114
GO:0016853	Isomerase activity	113
GO:0008135	Translation factor activity, nucleic acid binding	109
GO:0030246	Carbohydrate binding	106
GO:0003712	Transcription cofactor activity	99
GO:0016829	Lyase activity	98
GO:0008047	Enzyme activator activity	85
GO:0030695	GTPase regulator activity	68
GO:0016563	Transcriptional activator activity	67
GO:0016564	Transcriptional repressor activity	65
GO:0004386	Helicase activity	65
GO:0004803	Transposase activity	63
GO:0046906	Tetrapyrrole binding	62
GO:0003702	RNA polymerase II transcription factor activity	57
GO:0005200	Structural constituent of cytoskeleton	51
GO:0042277	Peptide binding	40
GO:0019207	Kinase regulator activity	39
GO:0019842	Vitamin binding	36
GO:0005201	Extracellular matrix structural constituent	36
GO:0003682	Chromatin binding	36
GO:0001871	Pattern binding	36
GO:0008307	Structural constituent of muscle	30
GO:0004601	Peroxidase activity	28
GO:0005212	Structural constituent of eye lens	26
GO:0019208	Phosphatase regulator activity	25
GO:0019825	Oxygen binding	24
GO:0008430	Selenium binding	23
GO:0003777	Microtubule motor activity	21
GO:0005496	Steroid binding	19
GO:0019239	Deaminase activity	18
GO:0051540	Metal cluster binding	18
GO:0043021	Ribonucleoprotein binding	13
GO:0008144	Drug binding	12
GO:0019840	Isoprenoid binding	10
GO:0042165	Neurotransmitter binding	9
GO:0005199	Structural constituent of cell wall	9
GO:0043028	Caspase regulator activity	7
GO:0042979	Ornithine decarboxylase regulator activity	6
GO:0015238	Drug transporter activity	6
GO:0030249	Guanylate cyclase regulator activity	6
GO:0030235	Nitric-oxide synthase regulator activity	5
GO:0008639	Small protein conjugating enzyme activity	5
GO:0003711	Transcriptional elongation regulator activity	5
GO:0030371	Translation repressor activity	4
GO:0009975	Cyclase activity	4
GO:0031406	Carboxylic acid binding	4
GO:0000146	Microfilament motor activity	4

GO, gene ontology; SGP, salmon genome project; TRAITS, TRanscriptome Analysis of Important Traits of Salmon.

* Number of features on TRAITS–SGP cDNA microarray with identified GO term.

### Dietary lipid experiment

The main findings of this microarray-based investigation will be reported in detail elsewhere. Here, data from preliminary analyses are presented to illustrate the degree of robustness and sensitivity that the microarray experimental design achieved.

The statistical analysis (two-way ANOVA – time and diet) identified 4142 features showing significant differential expression over the year-long experimental time course, demonstrating considerable temporal changes that may be related to a large number of biological factors. In contrast, only 15 significant diet-responsive features were detected (and an additional 10 features with significant time-point × diet interactions) Of the 15 significant diet-associated genes, 10 with BLASTX or other known sequence homologies had functions associated with either HUFA or cholesterol biosynthetic pathways (Table VI). The differential expression of these genes in these samples was confirmed by qRT-PCR. The identity of the remaining five of the 15 probes remains to be established. Calculation of the Pearson's correlation between microarray and qPCR fold-change data over four time-points, for the 11 probes analysed by both methods to date (including those detailed in this paper), gave a value of 0·81 (*P* < 0·001). This is indicative of a significant agreement between the two estimates of gene expression fold change.

**Table VI:** Two-way ANOVA *P* values (diet) and fold change comparisons for the probes and, or genes with known identity calculated from both microarray and quantitative real-time polymerase chain reaction (qRT-PCR) data. Values are the minimum and maximum recorded over the four time-points

			Fold change: VO *cf.* FO diet	
			
GAL file identifier	Diet *P* values	Identity/BLASTX hit	Microarray	qRT-PCR
hrt_opk_06O21	4·2 × 10^−06^	Lathosterol oxidase	0·4–1·4	1·2–2·9
bra_snb_04D02	2·3 × 10^−04^	Δ5 FAD (full-length EST)	1·5–2·7	
bra_bfo_03F11	8·5 × 10^−04^	Δ6 FAD (80% length, EST)	1·3–2·7	
can_D6O_S1B06	8·5 × 10^−04^	Δ6 FAD ORF	1·9–2·8	
can_D5O_S1B05	1·0 × 10^−03^	Δ5 FAD ORF	1·5–2·5	
can_D6D_S1B03	1·2 × 10^−03^	Δ6 FAD 3′-UTR (384 bp)	1·5–2·4	1·4–3·0
bra_snb_09B09	1·2 × 10^−03^	Squalene monooxygenase	0·2–1·3	1·7–6·3
bra_cbr_B4H01	9·6 × 10^−03^	Δ5 FAD (partial 3′-UTR, EST)	1·6–2·5	
can_D5D_S1B02	1·1 × 10^−02^	Δ5 FAD 3′-UTR (881 bp)	1·5–2·8	1·1–2·6
bra_opk_07F18	2·0 × 10^−02^	Isopentenyl-diphosphate Δ-isomerase 1	0·6–1·7	1·3–2·3

BLAST, basic local alignment search tool; EST, expressed sequence tag; FAD, fatty acyl desaturase; FO, fish oil; ORF, open reading frame; UTR, untranslated region; VO, vegetable oil.

To explore the consequences of analysing smaller numbers of microarrays, the significance of expression of the nine Δ5–Δ6 FAD probes present on the microarray was used as a proxy indicator for the performance of a given experimental design. Two strategies were employed to reduce microarray number: (a) omission of dye swap and (b) reduction of biological replicates. Two-way ANOVAs were undertaken (without multiple test correction) and gene lists ordered by ascending (diet) *P* value. The position of the nine probes in each of the lists is shown in Table VII. In a full analysis incorporating all six biological replicates and a dye swap (12 replicate microarrays per condition), the nine desaturase probes were all present in the top 25 of this list. Reducing microarray numbers, while retaining a dye swap, decreased apparent sensitivity but only markedly when the number of biological replicates was reduced to 3. At this replication level, the results of the analyses appeared to be sensitive to the particular biological replicates selected, as demonstrated by the duplicate analyses (biological replicates 1–3 *v.*4–6). Omission of a dye swap gave mixed results depending upon the dye selected for the pooled reference sample. With a Cy5 pooled reference, the results (all nine desaturase probes in top 27 of the list) closely matched those of the full 12 microarray design. However, with a Cy3 pooled reference, only three probes were in the top 25 of the list. One probe was not in the list at all and others had dropped as low as position 284.

**Table VII:** Sensitivity of microarray analysis to reduction in slide number. Figures in body of table refer to position of probe in two-way ANOVA gene lists ordered by ascending (diet) *P* value

	Dye swap	Yes	Yes	Yes	Yes	Yes	No (Cy5 reference)	No (Cy3 reference)

	Biological replicates	1–6	1–5	1–4	1–3	4–6	1–6	1–6

	Number of slides per condition	12	10	8	6	6	6	6

Gal file identifier	Feature identity							
bra_snb_04D02	Δ5 FAD (full-length EST)	4	5	11	26	1	4	9
can_D6O_S1B06	Δ6 FAD ORF (1400 bp)	5	6	5	8	42	8	8
bra_bfo_03F11	Δ6 FAD (80% length, EST)	6	10	14	40	3	5	87
can_D5O_S1B05	Δ5 FAD ORF (1400 bp)	7	8	10	21	4	23	7
can_D6D_S1B03	Δ6 FAD 3′-UTR (384 bp)	8	4	4	—	213	19	—
bra_cbr_B4H01	Δ5 FAD (partial 3′-UTR, EST)	10	12	13	18	31	9	26
can_D5D_S1B02	Δ5 FAD 3′-UTR (881 bp)	11	14	16	92	19	12	62
can_D6D_S1B04	Δ6 FAD 3′-UTR (401 bp)	16	34	6	7	451	14	284
can_D5D_S1B01	Δ5 FAD 3′-UTR (408 bp)	25	51	55	94	332	27	60

EST, expressed sequence tag; FAD, fatty acyl desaturase; ORF, open reading frame; UTR, untranslated region.

**Fig. 3:**
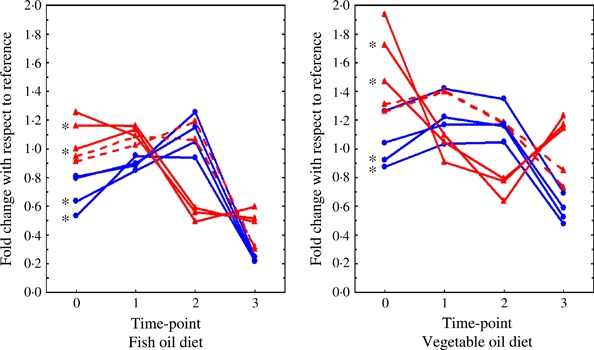
Fatty acyl desaturase (FAD) expression profiles (two diets over four time-points) derived from microarray analysis of nine different Δ5 FAD and Δ6 FAD probes. Blue lines denote Δ6 FAD probes, and red lines denote Δ5 FAD probes. Profiles for the four 3′-UTR probes are marked with an asterisk. Dashed red lines represent two Δ5 FAD probes [an open reading frame (ORF) PCR fragment and a full-length expressed sequence tag (EST) sequence], which show expression profiles that mimic those of the Δ6 FAD probes.

Expression profiles derived from the nine FAD probes over the four time-points and for both diets are depicted in Fig. 3. The four gene-specific 3′-UTR probes (*i.e.* two Δ5 and two Δ6 FAD fragments) show two distinct patterns of expression, which are also shared by the other desaturase probes on the array. However, while all Δ6 FAD probes show the same pattern of expression, two of the Δ5 FAD probes (a control ORF feature and a near full-length EST) show expression profiles that mimic those of the Δ6 FAD probes.

## Discussion

### TRAITS–SGP microarray fabrication

The TRAITS–SGP cDNA microarray was conceived as a preliminary tool, contributing towards the goal of developing a more focused DNA chip for routine health and performance monitoring in Atlantic salmon. A number of existing EST collections were used as the basis for its design and construction, and while this approach had obvious advantages in reducing the time frame and costs associated with resource development, it also made microarray fabrication all the more challenging. Not only did probe preparation involve a range of different host–vector combinations but also the need to track and annotate clones from different library resources compounded the difficulties. Most of the ESTs available comprised 5′-end reads. Although this improved the likelihood of successful probe annotation, use of mainly 5′-end sequence data will inevitably have compromised the cluster analysis as not all the clones will have been full length. The extent of gene redundancy on the microarray can only be confidently established from analysis of 3′-end sequence data. Presently, there are no plans to re-sequence the microarray resource.

Enriching the microarray for potential trait-specific genes has proven to be a worthwhile strategy. In a number of studies (Martin *et al.*, 2007; in prep.), SSH-derived probes have been identified as significant responders in immune, protein catabolism and smoltification studies. However, in lipid metabolism studies to date, no probes from diet SSH clones (or contigs containing SSH clones) have been identified as responding differentially between diets. One possible explanation for the apparent failure of the SSH procedure in this case follows from the subsequent microarray analysis of the dietary lipid experiment reported here. Overall fold changes in diet-responsive genes were found to be quite low, with the greatest changes being only three-fold, and there were no data to support differential expression of these genes at the two time points (50 and 53 weeks, *i.e.* pre- and post-smolt samples) used for diet SSH-library construction. HUFA biosynthesis in Atlantic salmon is known to vary during the growth cycle, with peak activity occurring around seawater transfer (Zheng *et al.*, 2005*b*).

There are no plans to radically improve upon the probe set in the TRAITS–SGP microarray. In its existing format, there is still spare capacity for feature printing, and additional candidate gene probes have been, and will be added on an *ad hoc* basis. For example, probes for 12 genes associated with lipid metabolism and 10 immune-related genes, not known to be on the microarray, have recently been included to facilitate two specific experimental studies. Over the past few years, printing technology has advanced significantly. Currently, the TRAITS–SGP microarray is being printed using non-contact inkjet-based technology (ArrayJet Ltd., Dalkeith, U.K.), which delivers more consistent spot and slide uniformity than contact pin printing and which should improve the reliability of generated microarray data and increase the sensitivity of detection.

### Dietary lipid experiment

By identifying candidate genes, the results of this experiment provide reassurance of the clone-tracking accuracy of the microarray. They also confirm that the selected microarray design and fabrication technology, together with the experimental methodology employed, provide the capacity for sensitive detection of differential expression. The fold change differences in expression of Δ5 FAD and Δ6 FAD between fish fed VO-based diets *v.* fish fed FO-based diets closely match those obtained by qRT-PCR analysis in a previous study (Zheng *et al.*, 2005*b*).

Microarray analyses are expensive and time consuming to perform, and there is often financial pressure to minimize the number of microarrays used in an experiment. Here, reducing the slide number from 12 to 8 (while retaining the dye swap) had little apparent effect on the ability to detect significant differential expression of FAD genes known to show a variable response based on diet. Using just six slides per condition (one per biological replicate and no dye swap) gave different outcomes according to the dye–target combination used. When the experimental sample was labelled with Cy3 and the pooled reference with Cy5, the results were comparable with the full 12 slide (including dye swap) analysis. However, there was much less agreement when the experimental sample was Cy5 labelled and the pooled reference was Cy3 labelled. The reason for this marked dye-dependent disparity remains to be established. However, it has been noted in this and other related studies conducted by TRAITS partners (and others) that (a) pooled reference samples produce higher background intensities and (b) the Cy3 channel consistently displays higher background values compared with the Cy5 channel. The combination of these factors may, at least in part, account for this phenomenon. The relevance of these observations to other studies is difficult to assess. The results are largely dependent on the homogeneity of the system under study and the absolute and relative expression levels of any differentially responding genes. For similar reasons, it is also difficult to critically assess published studies more generally. It is possible that the use of sex-balanced pools for experimental biological replication in this study, rather than individual samples, contributed to the discriminatory capabilities of this microarray experiment. Where cost considerations are an important factor, interrogation of reduced numbers of microarrays may be the only viable option available. If background fluorescence can confidently be controlled, omitting a dye swap, as opposed to reducing biological replication, may be a preferable route to follow in such cases.

The FAD expression profiles demonstrated the potential for obtaining artefactual results because of cross-hybridization of similar transcripts. Expression profiles derived from two Δ5 FAD probes (the ORF PCR fragment and full-length EST) closely matched those obtained from all Δ6 FAD probes. While the 3′-UTRs are very distinct (Δ5 FAD 3′-UTR = 1072 bp; Δ6 FAD 3′-UTR = 457 bp; sequence similarity *c.* 30%), the two ORFs are very similar to their Δ6 counterparts (both 1365 bp; *c.* 95% sequence similarity). It has also been reported from qRT-PCR analysis that Δ6 FAD gene expression is approximately four-fold higher than Δ5 FAD expression, in liver tissue of farmed salmon fed on FO (Zheng *et al.*, 2005*b*). Thus, it would appear that cross-hybridization of the more abundant Δ6 FAD transcript targets is masking the true expression profiles derived from these two Δ5 FAD probes. The failure of the microarray analysis to correctly distinguish between Δ5 FAD and Δ6 FAD expression profiles in all cases clearly illustrates one of the inherent weaknesses of cDNA microarray-based studies, *i.e.* the inability to distinguish between highly similar message transcripts. This is of particular relevance in interpreting transcriptomic data from salmonid species because fish of the family Salmonidae have undergone a relatively recent whole-genome duplication *c.* 25–100 million years ago (Allendorf & Thorgaard, 1984), such that simultaneous expression of duplicate genes is a commonly observed phenomenon.

### Other studies

As part of the original funded project, the TRAITS partners have already used the cDNA array to explore transcriptomic responses in experiments targeting the four key traits identified as being important for sustained salmon aquaculture, *i.e.* (a) supply of dioxin-free highly unsaturated oils for the salmon diet, (b) protein growth efficiency, (c) infectious disease and (d) a long and complex life cycle. In addition to the diet work outlined in this paper, other research at Stirling University, funded by the European Union, is comparing gene expression both within and between families of fish fed FO- or VO-based diets. Other studies, investigating the transcriptomic response of Atlantic salmon to infection by infectious pancreatic necrosis virus in both seawater and freshwater environments, are similarly in progress. Researchers at Aberdeen University are using the cDNA microarray to study the immune response in Atlantic salmon *in vivo* following infection by *A. salmonicida* and *in vitro* to examine the response to recombinant fish cytokines. Additionally, short-term starvation trials have been used to explore protein catabolism pathways. Finally, the Cardiff partner has identified genes and gene pathways from three tissues (brain, gill and kidney) that are involved in the parr–smolt transformation. The TRAITS–SGP microarray has also been supplied to an Australian research group to examine gene expression responses to amoebic gill disease (Wynne *et al.*, 2008). To date, its performance has not been critically assessed for use with other closely related species. Cross-species hybridization between salmonids has been shown to be extremely high (Rise *et al.*, 2004; von Schalburg *et al.*, 2005), suggesting that microarrays could be used with similar confidence for both Atlantic salmon and rainbow trout *Oncorhynchus mykiss*(Walbaum) because of the high level of sequence homology between the two species.

### Future directions

The final stage of the TRAITS project, *i.e.* development and validation of a focused oligonucleotide array, is currently in progress. Approximately 1000 differentially responding genes have been identified from cDNA microarray interrogations by the TRAITS partners and unique 70mer oligonucleotides designed and synthesized for these and appropriate control genes. These form the basis of the TRAITS–SGP secondary oligochip, the performance of which is currently being evaluated. Irrespective of the outcome, the TRAITS–SGP cDNA microarray will continue to be a valuable tool and be available for use to the wider scientific community. Enquiries regarding purchase and use of this microarray should be directed to ARK Genomics (http://www.ark-genomics.org) in the first instance.
